# Alterations of MicroRNAs in Solid Cancers and Their Prognostic Value

**DOI:** 10.3390/cancers2021328

**Published:** 2010-06-14

**Authors:** Panagiota Chira, Katerina Vareli, Ioannis Sainis, Christos Papandreou, Evangelos Briasoulis

**Affiliations:** 1 Human Cancer Biobank Center, University of Ioannina, University Campus, Ioannina 45110, Greece; E-Mails: panagiotachira@gmail.com (P.C.); kvareli@cc.uoi.gr (K.V.); isainis@cc.uoi.gr (I.S.); 2 Biomedical Research Institute, Foundation for Research & Technology, University Campus, Ioannina 45110, Greece; 3 Department of Biological Applications and Technologies, University of Ioannina, University Campus, Ioannina 45110, Greece; 4 School of Medicine, University of Thessaly, 22 Papakiriazi, Larissa 41222, Greece; E-Mail: cpapandreou@med.uth.gr (C.P.); 5 School of Medicine, University of Ioannina, University Campus, Ioannina 45110, Greece

**Keywords:** microRNA, prognostic biomarkers, cancer, solid tumors, gene-silencing, negative-regulation

## Abstract

MicroRNAs (miRNAs) are evolutionarily conserved, naturally abundant, small, regulatory non-coding RNAs that inhibit gene expression at the post-transcriptional level in a sequence-specific manner. Each miRNA represses the protein expression of several coding genes in a manner proportional to the sequence complementarity with the target transcripts. MicroRNAs play key regulatory roles in organismal development and homeostasis. They control fundamental biological processes, such as stem-cell regulation and cellular metabolism, proliferation, differentiation, stress resistance, and apoptosis. Differential miRNA expression is found in malignant tumors in comparison to normal tissue counterparts. This indicates that miRNA deregulation contributes to the initiation and progression of cancer. Currently, miRNA expression signatures are being rigorously investigated in various tumor types, with the aim of developing novel, efficient biomarkers that can improve clinical management of cancer patients. This review discusses deregulated miRNAs in solid tumors, and focuses on their emerging prognostic potential.

## 1. Introduction

MicroRNAs (miRNAs) are small, single-stranded RNA molecules. They are about 22 nucleotides (nt) in length and function as powerful gene expression regulators at the post-transcriptional level. In 1993, the first miRNA was identified within the roundworm, *Caenorhabditis elegans* [1]. Since this time, it has become evident that miRNAs are naturally abundant in both plants and animals [2]. Currently, over 720 human miRNAs have been identified. It is estimated that there may be approximately 1000 miRNA in the human genome (http://www.mirbase.org/). MiRNAs have broad regulatory potential and play important roles in diverse biological processes such as cell proliferation, apoptosis, metabolism, development, and differentiation [3]. Yet, miRNAs are expressed in a tissue- and development-specific manner. Changes in their expression have shown potential to correlate with disease status in various pathologic entities, such as Alzheimer’s disease, Parkinson’s disease, viral infections, diabetes, and myopathies. MiRNAs can also be used to identify tissues of origin in cancers [4,5,6]. A publicly available database “miR2Disease” (http://www.mir2disease.org/) documents known relationships between miRNA deregulation and human disease [7]. The present review focuses on cancer. Current published research suggests that aberrantly expressed miRNAs are involved in many molecular processes of cancer biology [8,9].

## 2. Evolution, Biogenesis, Function and Nomenclature

MiRNAs have been present since the dawn of animal life as small riboregulators. They have had an astonishingly slow, albeit very dynamic evolution. MiRNA development was recently shown to follow bilaterian evolution [10,11]. 

**Figure 1:**
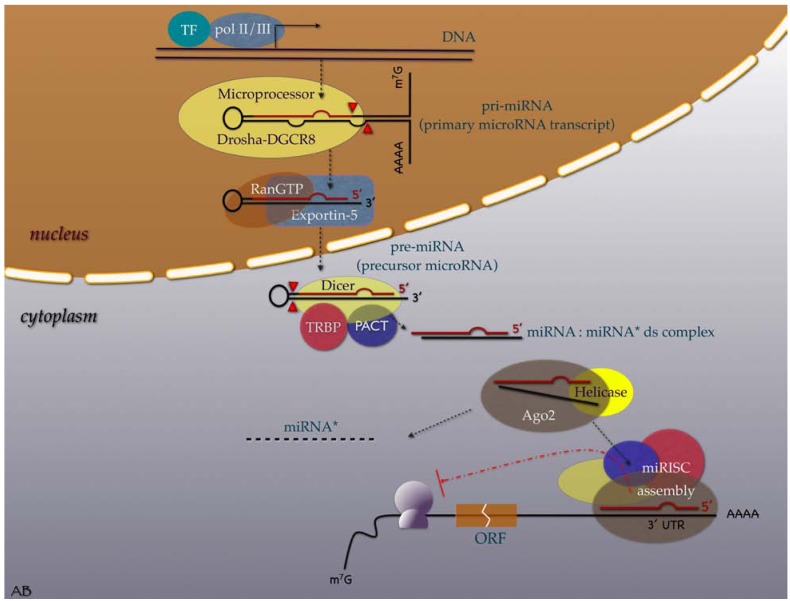
Illustrative overview of microRNA transcription, processing, maturation, and function in the human cell. (*Acronyms*: TF = transcription factor; pol = RNA polymerase; DGCR8 = CiGeorge critical region 8; RanGTP = Guanine triphosphatase (GTPase) Ran (RAs-related Nuclear protein); TRBP = Tar RNA binding protein; PACT = protein activator of PKR (Protein kinase R); ds = double strand; miRNA* = passenger microRNA; miRISK = microRNA induced silencing complex; Ago2 = Argonaute; AAAA = poly A tail; 3'UTR = three prime untranslated region; ORF = open reading frame; m^7^G = 7-methylguanosine cap. *Graphic symbols*: Yellowish oval shape symbolizes RNase enzymes; red line symbolizes the functional miRNA strand, red arrowheads indicate cleavage sites.

MiRNA biogenesis and function is illustrated in Figure 1. In humans, miRNAs are transcribed by RNA polymerase II or III as lengthy, primary microRNA (pri-miRNAs) that are hundreds of nucleotides (nt) long. These strands are capped and polyadenylated [12,13,14]. Pri-miRNAs are then endonucleolytically cleaved by the Drosha-DGCR8 microprocessor complex to generate a 70–90 nt long precursor miRNA (pre-miRNA), which then folds into an imperfect stem–loop hairpin structure [15]. These pre-miRNAs are transported to the cytoplasm by exportin-5, in complex with Ran-GTP [16], where they are further processed by Dicer to form transient 22 nt mature double stranded (ds) miRNA (miRNA:miRNA* duplexes). The functional strand of the duplex is preferably incorporated into a miRNA-associated RNA-induced silencing complex (miRISC) composed of Ago2, Dicer, TRBP, and PACT. The passenger strand separates to be degraded [17,18,19]. The mature miRNA guides the RISC to mRNAs that contain a sequence complementary to the miRNA seed-site. This site primarily specifies the mRNAs to be targeted [20,21]. MiRNAs regulate gene expression through one of two mechanisms, depending on the degree of miRNA complementarity with the target mRNA. When a miRNA binds to mRNA with perfect complementarity, it triggers its degradation by the RISC. Conversely, when a miRNA binds to imperfect complementary sites within the 3΄UTR of mRNA, it signals the inhibition of ribosomal translation (this mechanism is mainly observed in humans) [22].

Each miRNA gene is identified by a unique numerical name, which conveys limited information. The standard naming system uses abbreviated three letter prefixes to designate the species (e.g., hsa- in Homo sapiens, which is usually dropped in the literature), followed by the mir or miR three-letter tag connected to a number. The number is assigned sequentially by the miRBase Registry. The mature sequences are designated ‘miR’ (capitalized R), whereas the precursor hairpins are given ‘mir’. The same number indicates orthologous miRNAs (e.g., hsa-miR-101 in humans and mmu-miR-101 in mice). Lettered suffixes indicate paralogous sequences whose mature miRNAs differ at only one or two positions (e.g., miR-10a and miR-10b). Numbered suffixes indicate distinct hairpin loci that give rise to identical mature miRNAs (e.g., mir-281-1 and mir-281-2) [23]. 

## 3. MiRNAs in the Cancer Research Scene

The concept that miRNAs are involved in cancer pathogenesis as broad-spectrum riboregulators has revolutionized cancer research. Numerous studies on miRNAs and cancer have burst onto the scene over the last few years. MiRNAs appear to play key roles in almost all aspects of cancer biology, such as cell proliferation, apoptosis, invasion (metastasis), and angiogenesis [24]. The compelling hint of widespread miRNA deregulation in human cancer pathogenesis came from the analysis of the genomic distribution of 186 miRNA genes [9]. Calin *et al*. demonstrated that more than half of the miRNAs map to fragile or cancer-associated genomic regions that are prone to deletions, amplifications, or recombination. These aberrations can result in miRNA down- or upregulation, thus conferring selective advantages to mutated cells. Additional mechanisms of miRNA deregulation include altered expression of miRNAs as a consequence of excessive or deficient processing [25,26], aberrant transcription of the precursors by the epigenetic silencing of miRNA promoters [27,28] or as a result of the activity of oncogenic transcription factors [29,30], and rarely, point mutations in mature miRNAs or in target sequences that can interfere with normal target recruitment [31,32].

MiRNAs that are upregulated in cancers function as oncogenes, and miRNAs that are downregulated behave as tumor suppressors [33,34]. An archetypal oncogenic miRNA is miR-21. Using microarray analysis, miR-21 has been shown to be overexpressed in a wide variety of human cancers, including breast, colon, lung, and ovarian cancers, and in the glioblastoma multiform [34,35,36,37,38]. The overexpression of miR-21 has been shown to contribute to the malignant phenotype of glioblastoma by blocking the expression of critical apoptosis-related genes [37]. Additionally, miR-21 has been found to stimulate increased migration and invasion by hepatocellular carcinoma cells by inhibiting the expression of the phosphatase and tensin homolog (PTEN) tumor suppressor [39]. Thus, miR-21 represents an oncogenic miRNA, or ‘‘oncomir’’. In contrast, miR-15 and miR-16-1, by targeting BCL2 and freeing the apoptotic pathway, represent tumor-suppressive miRNAs [40]. MiR-34a is another example of a tumor-suppressive miRNA. Mir-34a is downregulated in one-third of colorectal cancers, and forms part of the p53 stress response pathway. Mir-34a is a direct transcriptional target of p53 and its overexpression after p53 activation mediates the key tumor suppressive effects of p53 through a context-dependent induction of growth arrest, apoptosis, or senescence [41,42,43]. After DNA damage in several types of cancer [44], silencing of miR-34a through aberrant CpG methylation of its promoter has been found to dominate over miR-34a’s transactivation by p53. 

Besides their active involvement in cancer initiation, several miRNAs have lately been shown to function in metastasis. Most data on this field originate from breast cancer research [45]. Characteristically, the first evidence came with the work of Ma *et al.*, who proved that the expression of miR-10b could initiate metastatic machinery in breast cancer by indirectly activating a well-characterized pro-metastatic gene, RHOC [46]. Moreover, it was recently demonstrated that the expression of the estrogen receptor (ER) is repressed by miR-206 and that a negative regulatory loop involving miR-221-222 and the ER may confer proliferative advantage and migratory activity to breast cancer cells and promote the transition from ER-positive to ER-negative tumors [47]. The documentation of the involvement of miRNAs in the regulation of cancer progression, metastasis, and treatment resistance opened up a new therapeutic challenge. Sequence-specific anti-miRNA oligonucleotides (antagomirs) have emerged as a novel class in cancer drug development [48]. Interestingly, the first published results have been very promising [49,50]. 

Several miRNA profiling platforms and techniques have been developed to assay known miRNA in biological samples [51]. Microarrays and miRNA-specific reverse transcription real-time polymerase chain reaction (RT-qPCR) are the two most often used for assessing miRNA expression profiles in human cancers. Microarrays rely on oligo-hybridization of non-amplified miRNAs contained in the test sample to specific probes preset on a chip's surface [52]. MiRNA-specific RT-qPCR approaches use either sequence-specific stem-loop primers (stem-loop RT-qPCR), or alternatively RNA poly-(A) polymerase to add a poly-(A) tail to the free 3’-hydroxyl end of mature miRNAs (poly-(A) tailing RT-qPCR) and sample amplification [53]. When compared, miRNA-specific RT-qPCR arrays were shown to outperform microarrays on reproducibility, sensitivity, and also specificity, which justifies their characterization as the gold-standard analytic approach in miRNA profiling in human [54]. Regarding cancer, none of the miRNA profiling techniques has shown cancer-type specificity. However, it must be stressed that sample-type specific pre-analytical steps should be taken seriously to avoid potential systematic biases in miRNA expression studies [55].

## 4. Deregulated miRNA Expression in Human Cancers

### 4.1. Cancers of the Respiratory System

#### Lung Cancer

MiRNA expression studies in human lung cancer have mostly focused on let-7, because the let-7 family members are highly expressed in the normal human lung and map to chromosome loci 3p22-23, which are usually lost in early lung cancers [56,57]. Takamizawa *et al*. investigated let-7 expression in surgically treated human lung cancers and found that let-7 expression was frequently reduced and was associated with poor prognosis. In the same study, the introduction of exogenous let-7 in the A549 lung adenocarcinoma cell line resulted in growth inhibition [58]. Others found that the expression of let-7 genes is 50% lower in cancerous lung tissue than that of healthy lung tissue. Low expression of let-7 also correlated with higher levels of expression of its target RAS protein [59]. Conversely, let-7g-expressing slow-growing NSCLCs were found to have reduced RAS and HMGA2 (high mobility group AT-hook 2) protein levels [60]. A few other miRNAs have also been identified as being underexpressed in lung cancer. Interestingly, the loss of miR-128b in NSCLC cell lines correlated with an increased production of the EGFR protein, suggesting that miR-128b is a key regulator of the EGFR protein [61]. Moreover, the miR-29 family members, miR-29a, mi-29b, and miR-29c, which are involved in the regulation of DNA methyltransferases, have been found to be downregulated in NSCLC [62]. Yu S. *et al*. identified a set of five miRNAs (low let-7a and miR-221, and high miR-137, miR-372, and miR-182) that predicted treatment outcome in NSCLC patients. Patients with a high risk score for these five miRNAs had an increased relapse rate and shortened survival times [63]. Finally, Hayashita *et al*. found that the miR-17-92 cluster, which is comprisesd of seven miRNAs, and which resides in intron 3 of the *C13orf25* gene at 13q31.3, is markedly overexpressed in lung cancers, especially with respect to small-cell lung cancer histology [64]. 

### 4.2. Breast Cancer

Several miRNA are differentially expressed in various tumor subtypes and clinical stages of human breast cancer. Typically, miR-206, which targets the estrogen (ER) receptor mRNA, is down-modulated in ER-positive breast cancers, and miR-30 is low within ER–negative tumors [65,66,67]. In contrast, miR-155, miR-213, and miR-203 were found to be overexpressed in advanced-stage breast cancers [67]. Moreover, upregulation of miR-373 and miR-520c can promote metastasis by inhibiting CD44 protein expression, while induction of miR-10b in otherwise non-metastatic breast tumors can initiate invasion and metastasis [46,68]. In addition, miR-21 has also been found to be upregulated in breast cancers that are associated with low expression of programmed cell death 4 (PDCD4) and tropomyosin 1 (TPM1) [69,70,71]. 

The deregulations of miRNAs that control the expression of genes encoding hormone-drug metabolizing enzymes are of particular interest in breast cancer. Tsuchiya *et al.* observed a significant inverse association between the expression levels of miR-27b and the CYP1B1 protein, which catalyzes the 4-hydroxylation of 17-estradiol. The expression level of miR-27b was decreased, while a high level of CYP1B1 protein was observed in breast cancer tissues, but not in the surrounding normal tissues [72]. Furthermore, miR-17–5p, also known as miR-91, has been found to be downregulated in breast cancer [73]. This miRNA normally represses the translation of the AIB1 (amplified in breast cancer 1) mRNA [73]. AIB1 is a co-activator of the cell cycle regulator E2F1, and also enhances estrogen receptor–dependent transcription [74,75]. In patients, the loss of expression of either miR-335 or miR-126 is associated with a shorter metastasis-free survival. An *in vivo* investigation showed that miR-126 restoration reduced overall breast cancer growth, whereas miR-335 could inhibit metastatic cell invasion by targeting the progenitor cell transcription factor SOX4 and the extracellular matrix component tenascin C, which has been correlated with tumor grade and patients’ survival [76,77].

### 4.3. Cancers of the Genital System

#### 4.3.1. Ovarian Cancer

In epithelial ovarian cancer, aberrant miRNA expression can differentiate normal *versus* cancer tissues. The most significantly overexpressed miRNAs identified in ovarian cancer are miR-200a, miR-141, miR-200c, and miR-200b, whereas miR-199a, miR-140, miR-145, and miR-125b1 are among the most down-modulated miRNA. Among these, the upregulated miR-200 family targets oncosuppressors BAP1and BRCA1, while miR-125 targets cMYC [35].

#### 4.3.2. Germ Cell Cancer

In an miRNA profiling study within malignant germ-cell tumors (GCT), miR-371-373 and miR-302 clusters were found to be universally overexpressed, regardless of the histological subtype, site (gonadal/extragonadal), or patient age [78]. These miRNAs have been shown to neutralize p53-mediated CDK inhibition, possibly through the direct inhibition of the expression of the tumor suppressor, LATS2. Therefore, the evidence suggests that these miRNAs act as oncogenes in the development of the human testicular germ cell tumors; they numb the p53 pathway and allow tumorigenic growth in the presence of wild-type p53 [79]. It has been suggested that specific sets of miRNA may also distinguish between different histological subtypes of testes tumors [80]. 

#### 4.3.3. Prostate Cancer

The mechanisms that underlie prostate cancer biology have been poorly defined. Recently, Ozen *et al*. discovered a widespread deregulation of miRNA expression in human prostate cancer, which implicates miRNA in prostate cancer. They observed downregulation of miR-125b, miR-145, and let-7c, which target genes whose products have been found to be increased in prostate cancer, such as RAS, E2F3, BCL-2, MCL-1, and EIF4EBP1 [81]. Another study on the miRNA profiling of prostate cancer detected the differential expression of 51 individual miRNAs between benign lesions and carcinoma tumors, the majority of which (72%, among which let-7, miR-16, -23, -92, 103, -125, -143, -145, -195, -199, -221, -222, and -497) were downregulated in carcinoma samples. Moreover, in the same study, differential miRNA expression showed the potential to classify carcinoma tumors according to their androgen dependence (hormone naive *versus* hormone refractory) [82].

### 4.4. Cancers of the Endocrine System

#### 4.4.1. Pituitary Cancer

MiR-15a and miR-16-1 levels have been found to be lower in pituitary adenomas when compared to that of normal pituitary tissue. Downregulation of these miRNAs in pituitary adenomas correlated with a greater tumor diameter and a lower p43 secretion (since the cofactors influence the activity of arginyl-tRNA-synthetase, which is involved in inflammation and angiogenesis), suggesting that these genes may, to some extent, influence tumor growth [83].

#### 4.4.2. Thyroid Cancer

Thyroid carcinomas are known to have numerous miRNA upregulated in comparison with unaffected thyroid tissue. Moreover, several miRNAs show the ability to serve as potent adjunct thyroid cancer class identifiers, especially with respect to distinguishing follicular thyroid carcinomas, papillary thyroid carcinomas, and anaplastic thyroid carcinomas [84]. Sheu *et al*. demonstrated that a set of five selected miRNAs could distinguish common variants of papillary thyroid carcinoma (PTC) from follicular adenoma (FA) and multinodular goiter. The mean values of the expression pattern of all miRNA in PTC showed a statistically significant difference from those in MNG and FA, by up to 90-fold for miR-146b [85]. Notably, most studies are in agreement that the three most upregulated miRNA in papillary thyroid carcinoma are miR-221, miR-222, and miR-146 [86].

### 4.5. Cancers of the Digestive System

#### 4.5.1. Hepatocellular Cancer

Expression profiling has shown that several miRNAs are deregulated in hepatocellular carcinoma (HCC) relative to normal liver tissue [87,88,89]. MiR-125b, which is known to suppress phosphorylation-activation of Akt, is usually downregulated in HCC and is associated with poor patient survival [89]. Jiang *et al*. profiled 200 precursor and mature miRNAs in fifty-four pairs of HCC tumors and adjacent benign liver tissue. They found that a large number of miRNA were upregulated in the cirrhotic and hepatitis-positive liver in comparison to the regulation in uninfected, noncirrhotic specimens. Additionally, several miRNAs were, on average, expressed at a higher level in benign liver tissues when compared with their expression in tumors. Moreover, they identified a set of 19 genes in HCC that significantly correlated with disease outcome; patients with good survival rates had overall higher mature miRNA expression levels compared with those that had poor survival rates [90]. In another study, miR-101 was found downregulated in 94% of tumor samples, compared with the regulation in matched adjacent noncancerous tissue. This indicates a potential application of missing miR-101 in the therapy of liver cancer [91].

MiR-221 and miR-21 are among the few upregulated miRNA in HCC. Direct targets of miR-221 include the cyclin-dependent kinase inhibitors (DDKI) CDKN1B/p27 and CDKN1C/p57, whose downregulation negatively affects HCC prognosis [92]. MiR-21, which is involved in the regulation of the PTEN tumor suppressor, was also found to be overexpressed in HCC lines, with a potency to drive cell growth, migration, and invasion [39]. Another study validated miR-224 overexpression in HCC and detected that miR-96 was overexpressed in HBV tumors, while miR-126 was downregulated in alcohol-related HCC [88].

#### 4.5.2. Colorectal Cancer

Several miRNAs are deregulated in colorectal cancer (CRC). Among them, a reduced expression of let-7, miR-143, miR-145, and miR-218, and higher expression of miR-155 have been found [34,93,94]. Moreover, in a miRNA microarray expression profiling study, high miR-21 expression in these tumors was identified as a potent predictor of poor survival and poor therapeutic outcome [95]. Nevertheless, of particular interest is a recent study on miR-135 that targets the adenomatous polyposis coli (APC) gene. Nagel *et al*. found considerable upregulation of miR-135 in colorectal adenomas and carcinomas, which correlated with low APC mRNA levels, regardless of the mutational status of APC. They suggested that their findings implicate a miRNA-mediated mechanism to colorectal cancer pathogenesis [96]. 

#### 4.5.3. Gastric Cancer

In gastric cancer, miR-21 is frequently found overexpressed and let-7, underexpressed [97,98]. Recent studies have shown that miRNAs are expressed differentially in gastric cancers, while specific miRNA signatures characterize histological subtypes. Definite miRs have a prognostic value. Low let-7g and high miR-214 were found to be associated with an unfavorable survival outcome that is independent of clinical covariates, including the depth of invasion, lymph-node metastasis, and stage [99]. Most recently, Tsujiura *et al*. found that plasma miRNA concentrations in patients with gastric cancers reflected the tumor tissue miRNA levels in most cases. In that study, the plasma concentrations of miR-17-5p, miR-21, miR-106a, and miR-106b were significantly higher in gastric cancer patients than in the controls, whereas let-7a was lower [100].

#### 4.5.4. Pancreatic Cancer

Pancreatic ductal adenocarcinoma (PDAC) is known for its grim prognosis. Bloomston *et al*. found that 15 overexpressed and eight underexpressed miRNAs could differentiate pancreatic cancer from chronic pancreatitis with 93% accuracy. Additionally, a high expression of miR-196a-2 was a predictor of poor survival [101]. Others have also identified that aberrant expression of miR-196a and miR-217 can discriminate PDAC from the samples of healthy pancreas, and from the pancreas of patients with chronic pancreatitis [102]. In addition, it has been shown that the analysis of miRNAs in pancreatic fine-needle aspirates can help to classify benign and malignant tissues [103]. 

### 4.6. Cancers of Central Nervous System

Expression profiling studies show that miR-21 is overexpressed and that miR-128-1 is commonly underexpressed in glioblastomas [104]. Oligodendrogliomas have a different miRNA expression profile characterized by high miR-9 and low miR-124 expression [105].

### 4.7. Head and Neck Cancers

Oral Squamous Cell Carcinoma

In a study of 102 cases of head and neck squamous cell carcinoma (HNSCC), of which 36% were HPV positive, a total of 42 miRNAs, including miR-1-2, miR-133a, and let-7d, showed lower expression levels relative to that of normal adjacent tissue; only miR-21 was steadily overexpressed [106]. A high expression of miR-21 was also found in the head and neck cancer cell lines [107]. In another study, miRNA-98, a possible regulator of the HMGA2 protein, was shown to be upregulated in these cancers [108]. 

### 4.8. Urinary System Cancers

#### 4.8.1. Kidney Cancer

MiRNA analysis in renal cell carcinomas has not yet provided a clear picture of the miRNA expression profile. In a small-sized study, a set of four human miRNA (miR-28, miR-185, miR-27, and let-7f-2) were found to be upregulated in a cohort of 20 renal cell carcinomas (P < 0.05) when compared to the expression within the normal kidney. Other studies observed a generalized downregulation of miRNA in these tumors [109,110]. In the case of Wilms’ tumors, miRNA expression was generally higher (miR-17-5p, miR-18a, miR-19b, miR-20a, miR-92) than that of normal renal parenchyma, or in other kidney tumor subtypes [111]. 

#### 4.8.2. Bladder Cancer

In a first study on bladder carcinomas, 10 miRNAs were found to be upregulated (miR-17-5, miR-23a-b, miR-26a-c, miR-103-1, miR-185, miR-203, miR-205, miR-221, miR-223) and miR-26 was found to be marginally downregulated [110]. However, a small recent study indicated that upregulation of miRNA in bladder tumor tissue is not a common feature [112].

### 4.9. Sarcomas

Human sarcomas constitute a heterogeneous group of over 50 different mesenchymal tumor subtypes, for which few diagnostic markers currently exist. In response to this clinical need, the expression profiles of miRNA in different sarcoma types were recently generated and led to the creation of a publically available web-based Sarcoma miRNA Expression Database S-MED (http://www.oncomir.umn.edu/SMED/index.php). S-MED is an open repository that describes the patterns of miRNA expression in various human sarcoma tumor types and in normal tissues [113].

#### 4.9.1. Soft Tissue Sarcomas

In the Kaposi sarcoma (KS), O’Hara *et al*. by using a precursor miRNA profiling method, found that loss of mir-221 and gain of mir-15 expression demarked the transition from immortalized to fully tumorigenic endothelial cells. Moreover, the expression of mir-140 and of KS–associated herpesvirus viral mRNAs increased linearly with the degree of transformation, while mir-24 emerged as a biomarker that was specific for KS [114].

Taulli *et al*. reported recently that the muscle-specific miR-1 and miR-206 are downregulated in rhabdomyosarcoma (RMS), a soft tissue sarcoma thought to arise from skeletal muscle progenitors. They showed that miR-1, which promotes myoblast differentiation, was barely detectable in the primary RMS of both the embryonal and alveolar subtypes, while for miR-206, the downregulation in tumors relative to that of normal muscle was less clear [115]. These two miRNA were found to suppress c-Met expression, while sub-functioning contributed to aberrant cell proliferation and to the migration of skeletal muscle cells, which leads to RMS development [116]. MiR-26a and miR-29 were also found to be under-presented in RMS. In all tumor samples, and in all cell lines, miR-26a was low, while its target Ezh2 (the histone methyltranferase, a known negative regulator of muscle differentiation) mRNA was higher than in controls. Similar findings apply to miR-29, an enhancer of myogenic differentiation, which functions as a regulatory myogenesis feedback with NF-κB and transcriptional repressor protein YY1 [117,118].

Furthermore, the miRNA expression profiles of 27 sarcomas, five normal smooth muscles, and of two normal skeletal muscle tissues were performed using microarray technology. The miRNA expression profiles were unique among the sarcoma types analyzed, and the miRNA expression signatures identified reflected the apparent lineage and differentiation status of each tumor type. The identification of unique miRNA signatures in each tumor type may indicate their significant role in tumorigenesis and may aid in the diagnosis of soft tissue sarcomas [119].

#### 4.9.2. Bone Sarcomas

MiR-34 was investigated in 117 primary osteosarcoma (OS) samples and was found to be decreased in tumor samples by means of deletions and epigenetic inactivation [120]. Downregulation of miR-34 seems to unleash its target genes, which are mainly those in the Notch pathway (DLL1, Notch 1 and Notch 2) that drive the metastatic molecular machinery in these tumors [121].

Another study showed that the expression of miR-1 and miR-206 were markedly decreased in both chordoma tissues and in cell lines when compared to expression in normal muscle tissue. Transfection of chordoma cell lines with miR-1 resulted in suppression of known miR-1 targets, such as Met and HDAC4, which are found to be overexpressed in chordoma [122].

### 4.10. Skin Cancers

Malignant Melanoma

Significant work has been done in profiling miRNA expression in Malignant Melanoma (MM). Micropthalmia-associated transcription factor (MITF), a ‘master regulator’ of melanocyte development and function, was the first gene identified as a target for miRNA-mediated regulation in melanoma. It was shown that miR-137 in melanoma cell lines downregulated MITF expression. The genomic locus of miR-137 at chromosome 1p22 places it in a region of the human genome that was previously determined to harbor an allele for melanoma susceptibility. Furthermore, a 15-bp variable nucleotide tandem repeat located immediately 5’ to the pre-mir-137 sequence has been shown to alter the processing and function of miR-137 in melanoma cell lines [123]. Similarly, upregulated miR-182 has been shown to stimulate the migration of melanoma cells *in vitro* and to enhance the cells’ metastatic potential *in vivo* by directly repressing MITF and FOXO3. In contrast, the downregulation of miR-182 impeded invasion and triggered apoptosis. MiR-182 expression also increased with the progression from primary to metastatic MM and was inversely correlated with FOXO3 and MITF levels [124]. 

The expression of miR-34a is silenced in a broad range of human tumors because of an aberrant CpG methylation of the corresponding promoter region. It is noticeable that 43.2% of the melanoma cell lines investigated, as well as 62.5% of the MM samples, showed methylation of the miR-34a promoter, which is not the case for normal primary melanocytes [44]. Another study demonstrated that miR-34b, miR-34c, and miR-199a behave as oncosuppressors in melanoma by targeting MET [125]. In addition, miR-34a was shown to inhibit uveal melanoma cell proliferation and migration through the downregulation of c-MET [126].

Members of the let-7 family of miRNAs were also significantly downregulated in MM as compared with their expression in benign nevi. This suggests their role as tumor suppressors in MM. Upregulation of let-7b in melanoma cells *in vitro* suppressed the expression of cyclins D1, D3, and A, and of cyclin-dependent kinase (CDK) 4. This inhibited cell cycle progression and anchorage-independent growth [127]. In addition, the loss of let-7a, which is a regulator of integrin β-3 expression, was shown to be involved in the development and progression of malignant melanoma [128]. Finally, Chen *et al*. investigated the expression of 470 miRNAs in tissue samples from benign nevi and metastatic melanomas. They found that miR-193b, a cyclin D1 regulator, represses cell proliferation and plays an important role in melanoma development [129].

## 5. Identified Prognostic miRNA

Accumulating evidence indicates that the expression of individual miRNA and miRNA signatures may become useful prognosticators in cancer. The discovery that miRNAs are remarkably stable molecules that can simultaneously regulate a multitude of protein coding genes prompted them to quickly be considered as unique molecular candidate biomarkers in cancer [100,130]. Thereafter, several research groups have embarked on discovering and validating miRNA expression signatures that have the potential to facilitate the molecular classification and to improve the prognostic information of human cancers [8]. Such miRNA signatures were first identified in leukemias [131,132] and recently in pediatric malignancies [133], in lung [63] and in cervical cancers [134]. Although such studies are yet to be considered mature enough for clinical usage, they indicate that soon, this new class of easily identifiable molecular biomarkers will be able to provide added help in advancing the therapeutic management of cancer by making the management more personalized [135]. 

**Figure 2:**
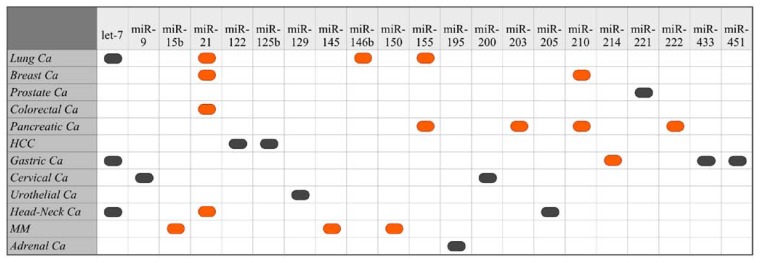
.Schematic overview of miRNAs identified as predictors of poor outcome in human cancers. Black rounded rectagles represent suppressed expression and orange rounded rectagles represent overexpression (HCC = hepatocellular carcinoma; MM = malignant melanoma).

Currently, a handful of microRNAs, which are graphically shown in Figure 2, have been identified as prognostic markers of cancer prognosis. 

### 5.1. Suppressed/Missing miRNAs as Biomarkers of Poor Cancer Prognosis

Most cancers are known to have lower miRNA levels than normal tissues, which indicates that downregulated miRNA could conceivably be considered potential cancer prognosticators [106,136,137]. Among all, let-7 has unequivocally been profiled as a cancer prognosticator in several studies. One of the first studies that investigated the prognostic value of tissue miRNAs provided strong evidence that let-7 expression is frequently reduced in lung cancer and is correlated with the poor survival of surgically treated patients [58]. This finding was confirmed by a second study, which also showed that a low expression of let-7a-2 in early stage lung adenocarcinoma correlated with poor prognosis [137]. Low let-7 was also found to be an indicator of a poor prognosis in gastric cancer [99] and in head and neck cancers [106].

Downregulated or missing miRNA have also been associated with poor prognosis in genitourinary cancers. In a large cohort study, prostate-specific miR-221 was found to be progressively suppressed in aggressive forms of prostate cancer. Spahn *et al*. conducted a microarray analysis of a set of 665 miRNAs in 92 cases of surgically treated prostate cancer. They found that the expression of miR-221 was significantly lower in prostate cancer when compared to the expression in non-malignant control tissue. This expression was progressively reduced in aggressive prostate cancer and metastasis, and had the potential to predict clinical recurrence in high-risk prostate carcinoma [138]. Another study identified low miR-129 as a prognosticator of disease progression in bladder cancer. The study also established a direct link between miR-129 and their two putative targets, GALNT1 (UDP-N-acetyl-alpha-dgalactosamine: polypeptide N-acetylgalactosaminyltransferase 1) and SOX4 (sex determining region Y-box 4), using luciferase assays [139]. In cervical cancer, Hu *et al*. analyzed tumor samples of 102 patients and identified that low miR-200a and miR-9 could predict patient survival. Based on this data, the investigators considered it reasonable to expect that the therapeutic delivery of miR-200a could be a potential treatment strategy for cervical cancer control [134].

In the clinical setting of adrenocortical tumors, it was shown that microarray profiling of miRNA could serve diagnostic purpose and could also provide prognostic information. Soon *et al*. found that miR-335 and miR-195 were significantly lower in adrenocortical carcinomas when compared with levels in adrenocortical adenomas, while low tumor expression of miR-195 could also identify a subset of adrenocortical carcinomas with a poor outcome [140]. 

Among cancers of the digestive system, deregulated miRNAs of prognostic value have been identified in HCC and gastric cancer. In HCC, low expression of miR-122 and MiR-125b characterized subsets of patients with a poor prognosis [89,141]. Two studies investigated the prognostic value of miRNAs in gastric cancer. Bandres *et al*. found that downregulation of miR-451 was associated with reduced disease-free and overall survival in gastric cancer patients [142]. Ueda *et al*. undertook a genome-wide miRNA expression profiling in two sets of gastric tissues and investigated for associations with the histological type, tumor progression, and prognosis of gastric cancer. In paired samples of non-tumor mucosa and cancer, they recognized an expression signature of 22 upregulated and 13 downregulated miRNA in gastric cancer, which could provide diagnostic information. Moreover, the low expression of let-7g and miR-433 was associated with an unfavorable outcome independently of clinical covariates, including depth of invasion, lymph-node metastasis, and stage [99].

Finally, in head and neck cancer, Childs *et al*. investigated miRNA expression in a cohort of 104 patients undergoing treatment with a curative intent. They found that low miR-205 in head and neck squamous cell carcinomas is significantly associated with locoregional recurrence independent of disease severity at diagnosis and treatment. Moreover, the combined low expression of let-7d and miR-205 was significantly associated with poor survival [106]. 

### 5.2. Overexpressed miRNAs as Biomarkers of Poor Cancer Prognosis

There is sound evidence that overexpressed miR-21, miR-146b, and miR-214 in cancer tissues constitute strong indicators of a poor prognosis, while a few others are projected as weak indicators.

Upregulated miR-21 has been identified as an indicator of poor prognosis in head and neck, colon, lung, and breast cancers. Jinsong Li *et al*. found that miR-21 is overexpressed in squamous cell carcinomas of the tongue relative to expression in adjacent normal tissues, and is a marker of poor survival [143]. Schetter *et al*. studied miRNA expression patterns of colon carcinomas and adjacent normal tissues in two independent cohorts of patients, a test cohort (84 patients from US) and a validation cohort (113 patients from Hong Kong). They investigated the clinical relevance of these patterns. A survival analysis of the pooled cohorts demonstrated that high miR-21 expression was associated with a poor prognosis in stage II or stage III patients, indicating its potential prognostic value [95]. Overexpressed miR-21 was also correlated with poor survival in early stage NSCLC [144] and in breast cancer. Yan *et al*. investigated, in a relatively small and heterogeneous cohort of breast cancer patients, the global expression profile of miRNAs in primary breast cancer and in normal adjacent tumor tissues for purpose of ascertaining the profile’s potential relevance to clinicopathological characteristics and to patient survival. They found an association of high miR-21 expression with a poor prognosis of breast cancer patients [145].

In lung cancer, Raponi *et al*. identified upregulated miR-146b as the most robust predictor of a poor prognosis of patients with squamous cell lung carcinoma [146]. Moreover, in another relatively small study, high miR-155 expression in primary tumors correlated with poor survival in early stage lung adenocarcinoma [137].

In gastric cancer, high expression of miR-214 was identified as a prognosticator of unfavorable outcomes in Japanese patients, independently of clinical covariates, including the clinical stage [99].

Camps *et al*. investigated the hypoxia-inducible miR-210 in tumor specimens and the patient outcome in 219 unselected breast cancer patients with a long-term follow-up of early breast cancer. They found that miR-210 expression levels showed an inverse correlation with disease-free and overall survival. The percentage of patients with a 10-year survival was 77% for those with miR-210 levels below the median and 53.6% for those with levels above the median [147]. 

In melanoma, Satzger *et al*. studied 16 miRNA with potential clinical significance in a small cohort of patients. They found that high expression of miR-15b was associated with a poor survival [148]. Most recently, Segura *et al*. identified a signature of 18 miRNA whose overexpression was significantly correlated with longer survival in patients with recurrent melanoma. Overexpressed miR-145 and miR-150 were the most prominent prognosticator miRNAs in that study [149]. 

Finally, in a cohort of 56 microdissected pancreatic ductal adenocarcinomas, significant correlations were found between elevated miRNA expression and overall survival for miR-155, miR-203, miR-210, and miR-222 [150]. 

## 6. Cancer Predisposition miRNA Polymorphisms

The notion that single nucleotide polymorphisms (SNPs) in protein-coding genes can affect the functions of encoded proteins and influence the individual susceptibility to cancers is well established. Currently, SNPs in miRNA genes, and also in their network genes (miRNA-binding sites, and miRNA processing machinery) is being investigated for cancer risk associations [151]. 

A recent case-control study of 1,009 breast cancer cases and 1,093 cancer-free controls in a population of Chinese women supported the idea that common SNPs in pre-miRNAs contribute to breast cancer susceptibility. In that study, the investigators Hu *et al*. found that mir-196a2 rs11614913:T4C and mir-499 rs3746444:A4G variant genotypes were associated with significantly increased risks of breast cancer [152]. In a similar study, Shen *et al*. identified a G to C polymorphism (rs2910164) within the sequence of the mir-146a precursor and demonstrated that a variant C allele led to increased levels of mature miR-146 in patients with breast and ovarian cancer and predisposed these patients to an earlier age of onset of familial breast and ovarian cancer [153]. It was also found that individuals with a common polymorphism in pre-mir-146a had decreased miR-146a expression and presented an increased risk of acquiring papillary thyroid carcinoma [154]. The above studies suggest that certain miRNA SNPs are associated with cancer susceptibility and indicate that research for cancer predisposition miRNA biomarkers is highly warranted.

## 7. Conclusions

The discovery of miRNAs has substantially changed our knowledge of gene expression regulation. In cancer, microRNAs emerge as novel blood- and tissue-based biomarkers for cancer detection, diagnosis, and prognosis, especially considering the limitations of currently available cancer tests [130,155,156]. However a great deal of work is required in this direction and many practical challenges remain to be addressed. This work includes validation of sensitive quantification assays, identification of miRNA target genes, and elucidation of the role of miRNAs in cancer molecular machinery. Currently, several studies have shown that cancers present characteristic alterations in miRNA expression. This data, considered together with the inherent stability of these tiny regulators of cell fate, suggest that miRNA assessment will probably evolve into a powerful tool for guiding personalized cancer management in the near future. 
